# Supplementary material for: Evolution of flowering time in a selfing annual plant: Roles of adaptation and genetic drift
Source: Ecol Evol. 2022 Jan 26;12(1):e8555. doi: 10.1002/ece3.8555 (PMC8794724; doi:10.1002/ece3.8555)
Supplement: Supplementary file 1 — Appendix S1 [file ECE3-12-e8555-s001.pdf]

Supplementary Information

Supplementary material. Supplementary material is available below and consist of the following. Table S1: List of the 17 populations sampled in Corsica (France). Table S2: Summary of the GLM on the sensitivity to vernalization. Table S3: Summary of the GLM on the relative seed production. Table S4: Genetic diversity at the 16 microsatellite loci. Table S5: Estimates of the selfing rate. Figure S1: Distribution of residual heterozygosity across MLGs for the two sampling years pooled. Figure S2: Distribution of MLGs in the population and through time. Figure S3: QQplot for the selection gradient in Figure 3B. Supplementary Material S1: Description of GENETHAPLO, a java program to analyse the genome-wide multilocus genetic structure of predominantly selfing or clonal populations. Supplementary Material S2: Details about the multiallelic method to simulate the effect of successive generations of drift. Supplementary Material S3: Comparison of the F_{ST} estimation variance when considering the loci as independent or using the multilocus genotypes as alleles of a single locus. Figure S4 and S5: results of the simulations.

Conflict of interest disclosure. The authors of this article declare that they have no financial conflict of interest with the content of this article.

Supplementary tables

Table S1. List of the 16 populations sampled in Corsica (France) with geographic coordinates and sampling years

Population	Latitude	Longitude	Altitude	Sampling years
FRA20025	42.756332397	9.4508333206	60	1985-2005
FRA20031	42.182167053	9.3766670227	130	1985-2005
FRA20035	42.361167908	9.4963331223	280	1985-2009
FRA20039	42.021499634	8.7348337173	410	1985-2005
FRA20043*	42.462001801	8.6848335266	40	1987-2009
FRA20044	42.551166534	8.7391662598	250	1985-2005
FRA20046	42.592441559	8.9075956345	240	1985-2005
FRA20049	42.444000244	9.4596662521	120	1985-2005
FRA20050	42.165782928	9.546872139	10	1985-2009
FRA20051	42.199165344	9.4646663666	100	1987-2005
FRA20056	41.41350174	9.1668329239	60	1985-2009
FRA20058	41.405334473	9.1265001297	195	1985-2009
FRA20069	42.591667175	8.9081668854	410	1987-2005
FRA20087	42.901668549	9.470000267	15	1987-2005
FRA20088	42.958332062	9.3950004578	160	1987-2005
FRA20089†	42.970500946	9.3668336868	380	1987-2009

Table S2. Effect of sampling year on sensitivity to vernalization, taking into account the family effect (genetic effect). For each effect, the variance component (with standard errors in brackets), the deviance, degrees of freedom, likelihood ratio (χ^2) and *p*-values are reported.

Tested effect on sensitivity to vernalization	Variance component (SE)	df	χ^2	p
block	0.01 (0.003)	1	21.0	5.10^{-6}
family	0.03 (0.008)	1	32.6	1.10^{-8}
error	0.13 (0.012)	542		

Table S3. Analysis of the family effect (genetic effect) on relative seed production (seed production standardized by year and treatment), taking into account the block effect. For each random effect, variance components (with standard deviations in brackets), degrees of freedom, likelihood ratio (χ^2) and *p*-values are reported.

Tested effect on relative seed production	Variance component (SD)	df	χ^2	p
block	0.024 (0.15)	1	116	$< 2.10^{-16}$
family	0.090 (0.30)	1	291	$< 2.10^{-16}$
error	0.153 (0.39)	1094		

Table S4. Genetic diversity at the 16 microsatellite loci for the Cape Corsica population in 1987 and 2009. n stands for the sample size, N_a and N_{a-rar} are the average number of alleles per locus and the allelic richness (after correction using a rarefaction method), H_e is the expected heterozygosity, F_{IS} is the heterozygote deficiency (both with 95% confidence interval in brackets, estimated by bootstrapping the individuals) and LD is the percentage of loci under significant linkage disequilibrium (for a type I error fixed at 5% when rejecting the equilibrium hypothesis). Multilocus diversity is described by n_{MLG}, the number of multilocus MLGs and n^h_{MLG}, the number of fully homozygous MLGs. Wilcoxon signed rank tests were performed across loci for N_{a-rar}, H_e and F_{IS} to test for a significant difference between the two years and the *p*-values are given.

Sampling year	n	N_a	N_{a-rar}	H_e (CI95)	F_{IS} (CI95)	LD	n_{MLG}	n^h_{MLG}
1987	64	3.6	3.4	0.351 (0.252-0.424)	0.942 (0.913-0.966)	89%	18	12
2009	81	3.9	3.7	0.623 (0.599-0.627)	0.967 (0.957-0.976)	96%	47	41
Total	145						60	48
p-value			0.211	6.10-5	0.090			

Table S5. Estimates of the selfing rate in the Cape Corsica population for each sampling year obtained using the program RMES by maximizing the log-likelihood of the whole multilocus heterozygosity structure of the sample. The 95% confidence intervals and the log-likelihood are given for the two unconstrained models and the constrained model, along with the *p*-value of the likelihood ratio test comparing constrained and unconstrained models.

Sampling year	Selfing rate [CI95]	Log-likelihood
(Unconstrained) 1987	0.944 [0.902-0.966]	-90.896
(Unconstrained) 2009	0.980 [0.974-0.986]	-84.592
Constrained	0.970 [0.960-0.978]	-177.952
p-value LRT		0.026

Supplementary Figures

Fig. S1. Distribution of residual heterozygosity across MLGs for the two sampling years pooled. Residual heterozygosity is defined here as the proportion of heterozygous loci in the multilocus genotype (over 16 loci) of each individual.

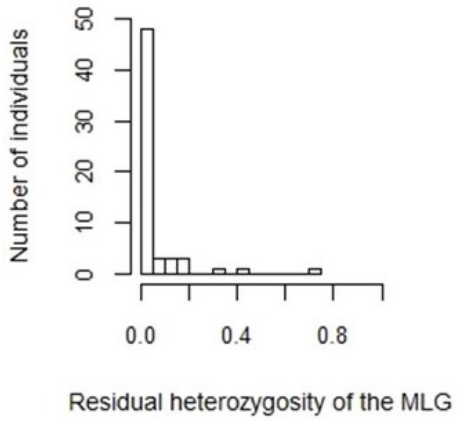

Fig. S2. Distribution of MLGs in the population and through time. The four most frequent MLGs are shared between years. MLGs with residual heterozygocity are shown in light grey (for the year 1987) and light green (for the year 2009).

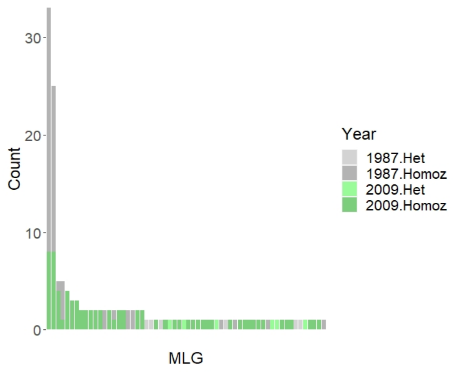

Fig. S3. Q-Q plots for the selection gradients shown on Figure 3B.

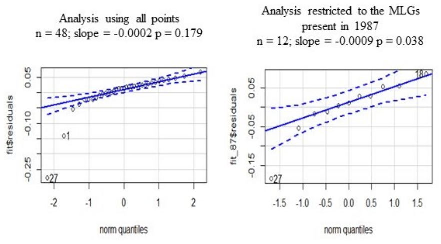

S1: GENETHAPLO: a java program to analyse the genome-wide multilocus genetic structure of predominantly selfing or clonal populations

Multilocus genotypes provide valuable information about mating systems (Jullien *et al.* 2019). Four software packages were previously developed to identify individuals originating from clonal reproduction using their multilocus genotype: MLGSIM (Stenberg *et al.* 2003); GENOTYPE and GENODIVE (Meirmans and Van Tienderen 2004), GENECLONE (Arnaud-Haond and Belkhir 2007) and poppr (Kamvar *et al.* 2014). Yet, none of these programs is specifically designed to identify individuals reproducing by selfing, in particular to detect repeated multilocus genotypes within a population and through time (or space) and recognize potential recombinants, formed by rare outcrossing events. GENETHAPLO is a program written in Java with four modules:

1. A module to convert the format of a dataset
2. A module to filter the dataset
3. A module to analyse the genetic diversity
4. A module to analyse the multilocus genetic structure

Formatting the data file. The first line of the data file is a header line describing the content of each column, i.e. the name of the population, of the sub-population, of the individual and of each locus. Each following line provides the genotype of an individual at the specified loci. The individuals should be sorted so that populations and sub-populations are grouped together in consecutive lines.

Example:

```
temp,pop,Individu,ENPB1,MTIC59L,MTIC37C,MTIC126,FMTBN,MTIC243,MTIC40,MTIC86
pop,1987,F20089-1987-001,278278,110110,086086,099099,198198,118118,128128,157157
pop,1987,F20089-1987-003,278278,110110,086086,099099,198198,118118,128128,157157
pop,1987,F20089-1987-004,278278,110110,086086,099099,198198,118118,128128,149149
...
pop,2009,F20089-2009-006,274280,097110,086086,099099,166166,118118,134134,126126
pop,2009,F20089-2009-007,278278,110110,086086,099099,198198,118118,128128,149149
pop,2009,F20089-2009-008,274280,097097,095095,099099,188188,118118,128128,155155
```

Module 1: format conversion. This module takes a dataset in the read2snp (Uricaru *et al.* 2014) format and converts it to a format suitable for GENETHAPLO, as detailed above.

Module 2: data filter. This module allows to filter out the loci, and individuals, having a percentage of missing data exceeding a specific threshold (given by the user). The two output files are i) a reduced dataset and ii) the list of the loci and individuals that have been removed. The percentage of missing data before and after filtering is also provided.

Module 3: genetic diversity. This module computes the key descriptors of genetic diversity classically used in population genetics studies. A first table summarizes the average number of individuals, alleles, the expected and observed heterozygosity and the F_{IS}. The selfing rate is also calculated from the F_{IS} for each sub-population. The module also provides these descriptors of diversity per locus and a table of allele frequencies for each sub-population.

Module 4: multilocus genetic structure. This module comprises three steps:

1. Grouping individuals according to their multilocus genotypes (thereafter called MLG): This module is based on a graph algorithm, where each node is an individual and nodes are connected when the individuals

share the same MLG. An error rate can be specified by the user to allow grouping MLGs that differ at less than a given proportion of loci. This avoids over-splitting the MLGs due to genotyping errors or recent mutations. The module also takes into account missing data that can generate uncertainties. In case of missing data, it is possible for an individual to have a genotype compatible with several MLG. In such a case, the individual is randomly assigned to one of the possible MLG groups based on a random draw where each MLG group has a probability of being chosen that is proportional to its size. The output files provide i) the list of all individuals with the MLG to which they are assigned ii) the list of all identified MLGs with their frequency in each sub-population, their residual heterozygosity, defined as the proportion of heterozygous loci out of the total number of loci without missing values, and the number of missing values in each MLG.

2. Estimating genetic distance between MLGs: The genetic distance between two MLGs is estimated as the number of alleles that differ between the two synthetic MLGs divided by the total number of alleles without missing data in these two MLGs. This module generates a distance matrix as well as a histogram depicting the pairwise distance distribution.

3. Identifying recombinant MLGs: This module uses the genetic distances to rapidly identify putative recombination events between MLGs. A MLG is a candidate recombinant between two other MLGs (thereafter called "parental MLGs") if the sum of the allele differences between it and its two putative parents equals the number of allele differences between these two parental MLGs. Only the MLGs that are represented by at least two individuals can be considered as potential parents. The output file provides a list of potential families, with the details of pairwise genetic distances.

Running the program. This java program can be launched from a command prompt, in the folder where the modules are stored, using the command `java -jar module.jar`, where `module.jar` should be replaced by the corresponding module name.

Module	Function	Module name	Arguments	Example
Module 1	format conversion	`atgcTol2_v1.jar`	infile	`java -jar atcgTol2 SNP_EPO_seuil_160.csv`
Module 2	data filter	`popFilter_v1b1win .jar`	infile<br>+ threshold of missing data for individuals and loci	`java -jar popFilter_v1b1win.jar F20089.csv 25 35`
Module 3	genetic diversity	`popDiversity_v1b1 _win.jar`	infile	`java -jar popDiversity_v1b1_win.jar F20089.csv`
Module 4	multilocus genetic structure	`genetHaplo_v1b1.j ar`	infile<br>+ error rate<br>+ type of analysis*<br>+ random seed	`java -jar atcgTol2 F20089.csv 5 d 0`

* the type of analysis for the module 4 can be:
- only MLG groups (no argument)
- MLG groups and distances (`d` as an argument, as shown in the example)
- MLG groups + distance + potential recombinants (`r` as an argument, as shown in the example)

If no argument (infile or option) is added in the command, a short description of the script is displayed.

For example:

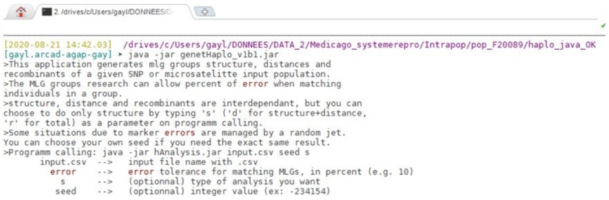

```
2. /drives/c/Users/gayl/DONNEES/D
[2020-08-21 14:42.03]  /drives/c/Users/gayl/DONNEES/DATA_2/Medicago_systemerepro/Intrapop/pop_F20089/haplo_java_OK
[gayl.arcad-agap-gay] ➤ java -jar genetHaplo_v1b1.jar
>This application generates mlg groups structure, distances and
recombinants of a given SNP or microsatelitte input population.
>The MLG groups research can allow percent of error when matching
individuals in a group.
>structure, distance and recombinants are interdependant, but you can
choose to do only structure by typing 's' ('d' for structure+distance,
'r' for total) as a parameter on programm calling.
>Some situations due to marker errors are managed by a random jet.
You can choose your own seed if you need the exact same result.
>Programm calling: java -jar hAnalysis.jar input.csv seed s
       input.csv  -->   input file name with .csv
         error    -->   error tolerance for matching MLGs, in percent (e.g. 10)
           s      -->   (optionnal) type of analysis you want
          seed    -->   (optionnal) integer value (ex: -234154)
```

Example of output of the console:

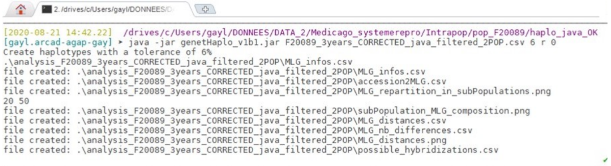

```
2. /drives/c/Users/gayl/DONNEES/D
[2020-08-21 14:42.22]  /drives/c/Users/gayl/DONNEES/DATA_2/Medicago_systemerepro/Intrapop/pop_F20089/haplo_java_OK
[gayl.arcad-agap-gay] ➤ java -jar genetHaplo_v1b1.jar F20089_3years_CORRECTED_java_filtered_2POP.csv 6 r 0
Create haplotypes with a tolerance of 6%
.\analysis_F20089_3years_CORRECTED_java_filtered_2POP\MLG_infos.csv
file created: .\analysis_F20089_3years_CORRECTED_java_filtered_2POP\MLG_infos.csv
file created: .\analysis_F20089_3years_CORRECTED_java_filtered_2POP\accession2MLG.csv
file created: .\analysis_F20089_3years_CORRECTED_java_filtered_2POP\MLG_repartition_in_subPopulations.png
20 50
file created: .\analysis_F20089_3years_CORRECTED_java_filtered_2POP\subPopulation_MLG_composition.png
file created: .\analysis_F20089_3years_CORRECTED_java_filtered_2POP\MLG_distances.csv
file created: .\analysis_F20089_3years_CORRECTED_java_filtered_2POP\MLG_nb_differences.csv
file created: .\analysis_F20089_3years_CORRECTED_java_filtered_2POP\MLG_distances.png
file created: .\analysis_F20089_3years_CORRECTED_java_filtered_2POP\possible_hybridizations.csv
```

Example of output figures:

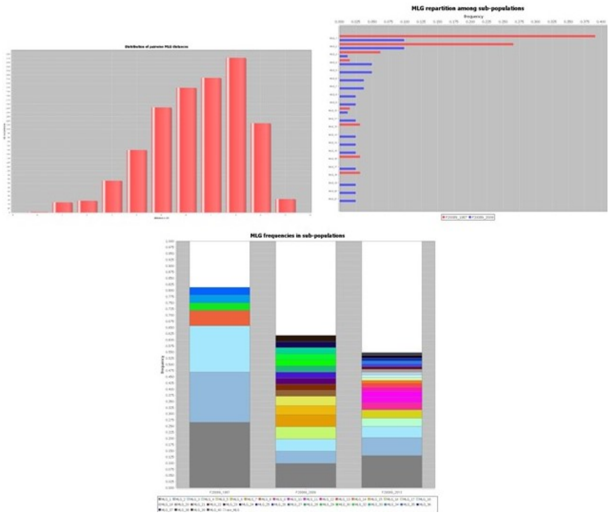

GENETHAPLO is a program freely available at `https://github.com/laugay/GenetHaplo`. Source codes are available from authors upon request.

References. Arnaud-Haond, S., and K. Belkhir. 2007. genclone: a computer program to analyse genotypic data, test for clonality and describe spatial clonal organization. Molecular Ecology Notes 7:15-17.

Jullien, M., M. Navascués, J. Ronfort, K. Loridon, and L. Gay. 2019. Structure of multilocus genetic diversity in predominantly selfing populations. Heredity 123:176-191.

Kamvar, Z. N., J. F. Tabima, and N. J. Grünwald. 2014. Poppr: an R package for genetic analysis of populations with clonal, partially clonal, and/or sexual reproduction. PeerJ 2:e281-e281.

Meirmans, P. G., and P. H. Van Tienderen. 2004. genotype and genodive: two programs for the analysis of genetic diversity of asexual organisms. Molecular Ecology Notes 4:792-794.

Stenberg, P., M. Lundmark, and A. Saura. 2003. mlgsim: a program for detecting clones using a simulation approach. Molecular Ecology Notes 3:329-331.

Uricaru, R., G. Rizk, V. Lacroix, E. Quillery, O. Plantard, R. Chikhi, C. Lemaitre, and P. Peterlongo. 2014. Reference-free detection of isolated SNPs. Nucleic Acids Research 43:e11.

S2: Details about the multiallelic method to simulate the effect of successive generations of drift

We simulated the effect of 22 generations of drift, using an extension to multiallelic data of the approach described in Frachon *et al.* (2017) and inspired by Goldringer and Bataillon (2004). In order to account for the sampling variance in initial MLG frequencies, we simulated individual MLG frequency trajectories as follows: suppose that we observe a vector $\mathbf{y}$ of MLG counts, out of n total counts, in the 1987 sample. We assume that these observed counts are drawn from a multinomial distribution $\text{Mult}(n, \mathbf{x})$ where $\mathbf{x}$ is the vector of (unknown) MLG frequencies in the 1987 population. Assuming a Dirichlet $\text{Dir}(1)$ prior distribution for $\mathbf{x}$, and using the Bayes inversion formula, the posterior distribution of $\mathbf{x}$ is distributed as $\text{Dir}(\mathbf{y}+1)$. For each simulation, we therefore randomly draw the initial MLG frequencies in the 1987 sample $\boldsymbol{\pi}_{1987}$, from a $\text{Dir}(\mathbf{y}+1)$ distribution. We then draw "pseudo-observed" MLG counts using a random draw from $\text{Mult}(n, \boldsymbol{\pi}_{1987})$.

References. Frachon, L., C. Libourel, R. Villoutreix, S. Carrère, C. Glorieux, C. Huard-Chauveau, M. Navascués, L. Gay, R. Vitalis, E. Baron, L. Amsellem, O. Bouchez, M. Vidal, V. Le Corre, D. Roby, J. Bergelson, and F. Roux. 2017. Intermediate degrees of synergistic pleiotropy drive adaptive evolution in ecological time. Nature Ecology Evolution 1:1551-1561.
Goldringer, I., and T. Bataillon. 2004. On the distribution of temporal variations in allele frequency: consequences for the estimation of effective population size and the detection of loci undergoing selection. Genetics 168:563-568.

S3: Comparison of the F_{ST} estimation variance when considering the loci as independent or using the multilocus genotypes as alleles of a single locus

Due to reduced effective recombination, the entire genome of a predominantly selfing population can behave as a giant supergene. This violates the hypothesis of independence between loci that is commonly assumed in population genetics, in particular for F_{ST} estimation. One solution to this violation could be to take the linkage disequilibrium into account by concatenating all loci and considering the distinct multilocus genotype (thereafter MLG) as different alleles of a single (mega) locus. Here we use simulations to compare estimates of genetic differentiation measured using all loci considered independent or using MLGs as alleles of a single locus, and compare bias and estimation variance (MSE).

Methods. We used the individual-based simulations of diploid hermaphroditic populations performed using SLiM 2.5 (Haller and Messer 2017) by Jullien *et al.* (2019). Briefly, we simulated the evolution of 20 independent loci (with a recombination rate of 0.5) in an isolated population with a given selfing rate and effective population size. Each simulation comprised two periods. A first period of 25 N generations (with N the demographic population size, measured as the number of diploid individuals) allowed the populations to reach the mutation-drift equilibrium. At this stage (time t_0 = 0), 100 diploid individuals were randomly sampled. Twenty generations later (t_{20}), a second sample of 100 individuals was drawn. This matches the sampling design performed on the focal population in Cape Corsica. Further details can be found in Jullien *et al.* (2019). We simulated 1,000 replicates for populations with a selfing rate ranging between 0.8 and 1 and a population size N of 250 individuals. For each simulated dataset, we assessed the relative temporal differentiation between the two samples using Weir and Cockerham's (1984) estimator of F_{ST}, as implemented in the R package hierfstat (Goudet 2005). We then grouped individuals with identical combinations of alleles (multilocus genotypes, MLG) using the program GENETHAPLO (Supplementary Material S1 above and as detailed in the main text, except for the error rate, which was set to zero). MLGs with residual heterozygosity were removed for the multilocus assessment of temporal differentiation. We considered each MLG as an allele of a single locus and computed the allele frequency for each temporal sample (t_0, t_{20}). We used the function haploDiv of the R package diversity (Keenan *et al.* 2013) to estimate the F_{ST} on this haploid locus using Weir and Cockerham's method (1984). We also reiterated this analysis without removing the MLGs with residual heterozygosity to assess the effect of this step on the bias and variance of F_{ST} estimation.
For each simulated selfing rate, we calculated the expected F_{ST} using the relationship established in Frachon *et al.* (2017): $F_{ST} = \tau/(4N_e + \tau)$, where N_e is the number of haploid individuals (or gene copy number) and τ the number of generations between the two temporal samples. Selfing reduces the number of independent gametes sampled for reproduction, so that the effective size is reduced to $N_e \sim N(2-\sigma)/2$ (Wright 1969, Pollak 1987) with σ the selfing rate and N the population size. As a result, $F_{ST} = \frac{\tau}{4N\frac{2-\sigma}{2}+\tau}$.
We measured the bias as the difference between this reference F_{ST} and the F_{ST} we estimated assuming independent loci or the F_{ST} we estimated using MLGs as alleles of a single locus. We measured the mean square error as the sum of the bias and the variance: $MSE = \sum_{i=1}^{1000}(\widehat{F_{ST\ indep\ or\ MLG}}(i) - F_{ST\ expected}(i))^2$.

Results and discussion. When using MLGs as alleles of a single locus, the estimated F_{ST} suffers from an increased negative bias compared to the method assuming that all loci are independent (Fig. S4). The bias decreases with increasing selfing rate but is always neg-

ative for the selfing rates we considered (>0.8), which will tend to overestimate the effective population size (Fig. S4). This bias is likely due to the dependence of F_{ST} on the frequency of the most frequent allelic type (Jakobsson *et al.* 2013, Edge and Rosenberg 2014, Alcala and Rosenberg 2017): as the number of alleles increases, the frequency of the most frequent allele necessarily decreases, which sets an upper bound to the F_{ST} estimates (Fig. 2 in Jakobsson *et al.* 2013). Removing MLGs with residual heterozygosity reduces the bias, because heterozygous MLGs are generally unique and therefore form new alleles of the single "MLG" locus. In addition, the precision of the F_{ST} estimates using the MLG method is expected to decrease when the number of loci considered increases, because genetic diversity is influenced by haplotype length (Mehta *et al.* 2019). As already shown (Navascués *et al.* 2020), the variance of the F_{ST} estimation assuming independent loci increases with high selfing rates (Fig. S5), due to the linkage disequilibrium that reduces the number of effective loci (Golding and Strobeck 1980, Nordborg 2000). Surprisingly, the MLG method seems to limit the estimation variance. This is most probably artificial, because the upper-bound on the F_{ST} estimates also constrains its variance. Altogether, despite the high sampling variance due to the low number of effective loci available under strong selfing, our results suggest that it is preferable to assume that all loci are independent instead of using MLGs to estimate F_{ST}.

Fig. S4. Estimates of temporal differentiation (F_{ST}) using all loci and assuming independence (in black) or using the MLG (concatenated genotype) as alleles of a single locus, with (green) or without (blue) exclusion of the MLGs with residual heterozygosity. The red line stands for the expected value for the F_{ST} where $F_{ST} = \frac{\tau}{4N\frac{2-\sigma}{2}+\tau}$ with τ the number of generations between the two temporal samples, σ the selfing rate and N the simulated population size.

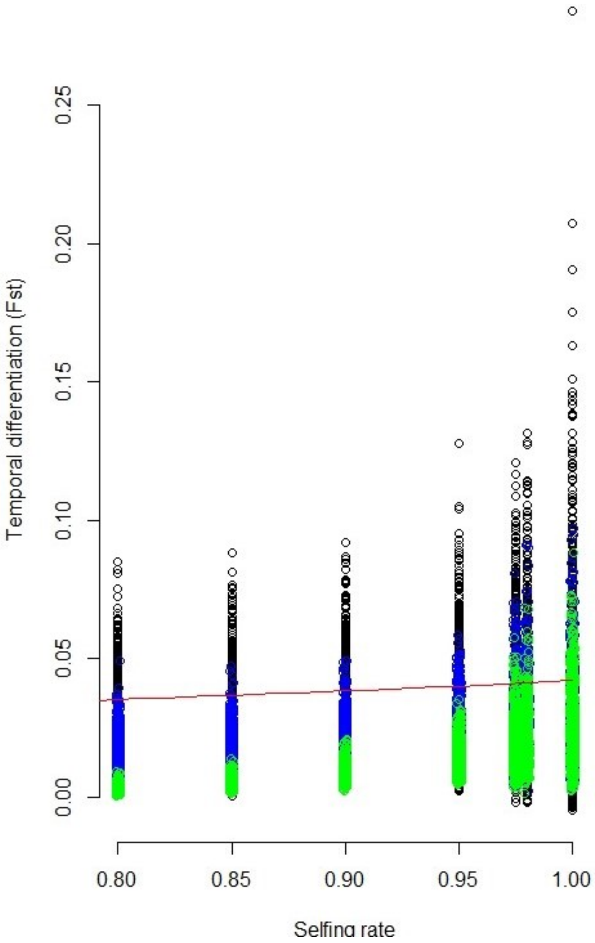

Fig. S5. Average bias (A) and MSE (B) for the estimation of temporal differentiation (F_{ST}) using all loci and assuming independence (in black) or using the MLG (concatenated genotype) as alleles of a single locus, with (green) or without (blue) exclusion of the MLGs with residual heterozygosity.

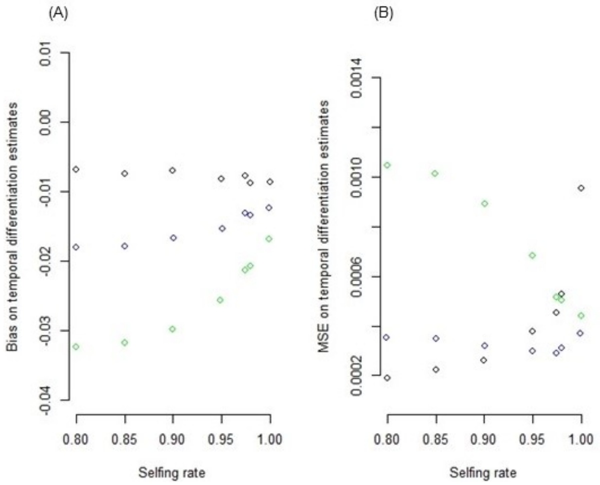

References. Alcala, N., and N. A. Rosenberg. 2017. Mathematical Constraints on F_{ST}: Biallelic Markers in Arbitrarily Many Populations. Genetics 206:1581-1600.

Edge, M. D., and N. A. Rosenberg. 2014. Upper bounds on F_{ST} in terms of the frequency of the most frequent allele and total homozygosity: The case of a specified number of alleles. Theoretical Population Biology 97:20-34.

Frachon, L., C. Libourel, R. Villoutreix, S. Carrère, C. Glorieux, C. Huard-Chauveau, M. Navascués, L. Gay, R. Vitalis, E. Baron, L. Amsellem, O. Bouchez, M. Vidal, V. Le Corre, D. Roby, J. Bergelson, and F. Roux. 2017. Intermediate degrees of synergistic pleiotropy drive adaptive evolution in ecological time. Nature Ecology Evolution 1:1551-1561.

Golding, G. B., and C. Strobeck. 1980. Linkage disequilibrium in a finite population that is partially selfing. Genetics 94:777-789.

Goudet, J. 2005. Hierfstat, a package for R to compute and test hierarchical *F*-statistics. Molecular Ecology Notes 5:184-186.

Haller, B. C., and P. W. Messer. 2017. SLiM 2: Flexible, interactive forward genetic simulations. Molecular Biology and Evolution 34:230-240.

Jakobsson, M., M. D. Edge, and N. A. Rosenberg. 2013. The relationship between F_{ST} and the frequency of the most frequent allele. Genetics 193:515-528.

Jullien, M., M. Navascués, J. Ronfort, K. Loridon, and L. Gay. 2019. Structure of multilocus genetic diversity in predominantly selfing populations. Heredity 123:176-191.

Keenan, K., P. McGinnity, T. F. Cross, W. W. Crozier, and P. A. Prodöhl. 2013. diveRsity: An R package for the estimation and exploration of population genetics parameters and their associated errors. Methods in Ecology and Evolution 4:782-788.

Mehta, R. S., A. F. Feder, S. M. Boca, and N. A. Rosenberg. 2019. The relationship between haplotype-based F_{ST} and haplotype length. Genetics 213:281-295.

Navascués, M., A. Becheler, L. Gay, J. Ronfort, K. Loridon, and R. Vitalis. 2020. Power and limits of selection genome scans on temporal data from a selfing population. bioRxiv:2020.2005.2006.080895.

Nordborg, M. 2000. Linkage disequilibrium, gene trees and selfing: An ancestral recombination graph with partial self-fertilization. Genetics 154:923-929.
Pollak, E. 1987. On the theory of partially inbreeding finite populations .1. Partial selfing. Genetics 117:353-360.
Weir, B. S., and C. C. Cockerham. 1984. Estimating F-statistics for the analysis of population structure. Evolution 38.
Wright, S. 1969. Evolution and the Genetics of Populations. Vol. II. The Theory of Gene Frequencies. University of Chicago Press, Chicago.
